# Optimization of abamectin production by *Streptomyces avermitilis* and its antagonistic activity against *Meloidogyne incognita*

**DOI:** 10.1186/s12896-026-01112-6

**Published:** 2026-03-11

**Authors:** Mona S. Zayed, Ahmed A. M. Abdelhafez, Ahmed E. Mahgoub, Olfat Radwan, Wafaa H. Radwan

**Affiliations:** 1 Department of Microbiology, Faculty of Agriculture, Ain Shams University, P.O. Box 68, Hadayek Shoubra, Shoubra El-Kheima, Cairo, 11566 Egypt; 2 Department of Plant Protection, Faculty of Agriculture, Ain Shams University, P.O. Box 68, Hadayek Shoubra, Shoubra El-Kheima, Cairo, 11566 Egypt; 3 Faculty of Organic Agriculture, Heliopolis University for Sustainable Development, Cairo, Egypt; 4 Pesticides Research Analysis Department, Central Agricultural Pesticides Laboratory, 7 Nadi El-Seid St, Giza, Dokki 12618 Egypt

**Keywords:** Abamectin, *Streptomyces avermitilis*, *Meloidogyne incognita*, Nematicidal activity, Eco-friendly agent, Secondary metabolite production

## Abstract

This study aims to evaluate the safety of *Streptomyces avermitilis* and its extracts, as well as the impact of temperature, pH, and salinity on its growth, nematicidal activity, and abamectin production. It also determines the optimal conditions that promote significant mycelium and abamectin yield. *S. avermitilis*’ extract was found to be non-toxic to human skin fibroblasts and certain selected microorganisms. The highest mycelium dry weight and nematicidal effectiveness (99%) were achieved at pH 7 after 8 days of incubation at 28 °C, with a weight of 6.45 g/L at 0.1% NaCl and 7.81 g/L at 1% NaCl. The production medium supported significantly the highest mycelium production (23.57 g/L) and abamectin yield (3.23 g/L) as well as the highest specific growth rate (µ = 0.65/d), and multiplication rate (MR) (0.93) and the lowest doubling time t_d_ (1.06 d). Taguchi design screened seven variables, and identified potato starch, α-amylase, and incubation period as significant factors (*p* ≤ 0.05). The Box-Behnken design yielded maximum mycelium (71.08 g/L) and abamectin production (8.30 g/L) under optimal conditions of 150 g/L potato starch, 0.15 g/L α-amylase, and a 10-day incubation period. This investigation demonstrates the strong potential of *S. avermitilis* as an eco-friendly biocontrol agent against *M. incognita* and highlights the power of response surface methodology in optimizing secondary metabolite production for commercial and agricultural applications.

**Supplementary Information:**

The online version contains supplementary material available at 10.1186/s12896-026-01112-6.

## Introduction

Abamectin is a naturally occurring macrocyclic lactone produced by the soil bacterium *Streptomyces avermitilis.* It is widely used in agriculture and veterinary medicine because of its strong insecticidal and anthelmintic activity combined with relatively low toxicity to higher mammals [1–3]. Owing to increasing restrictions on synthetic chemical pesticides and the growing demand for sustainable agricultural practices, abamectin has gained renewed attention as an environmentally compatible component of integrated pest management programs [4].

One of the most important agricultural applications of abamectin is the control of plant-parasitic nematodes, particularly root-knot nematodes (*Meloidogyne* spp.), which cause substantial yield losses across a wide range of economically important crops [5]. Using abamectin as a biocontrol agent provides an effective alternative to conventional chemical nematicides, offering high efficacy as well as reducing adverse impacts on non-target organisms and the environment [6, 7].

The biosynthesis of abamectin in *S. avermitilis* is strongly influenced by nutritional composition and environmental conditions that regulate mycelial growth and secondary metabolite formation. Carbon source, enzymatic supplementation, pH, temperature, salinity, and incubation period are known to affect production efficiency. Consequently, optimizing fermentation conditions is essential to enhance abamectin yield and improve the economic feasibility of large-scale production [8, 9].

Traditional optimization approaches, such as “one-factor-at-a-time,” are inadequate for complex microbial systems because they fail to capture interactions among multiple variables and require extensive experimentation. In contrast, statistical optimization techniques, such as response surface methodology (RSM), enable simultaneous evaluation of multiple factors and their interactions, allowing efficient identification of optimal fermentation conditions while reducing experimental cost and time [10]. Although RSM has been used to improve the production of various microbial secondary metabolites, its integrated application to abamectin production under combined nutritional and environmental constraints remains limited [11, 12].

In addition to production efficiency, the safe application of abamectin requires careful evaluation of its biological selectivity. Although abamectin is widely regarded as an eco-friendly compound, systematic assessment of its effects on beneficial soil microorganisms and human-relevant cell models remains insufficiently addressed in studies focused on production optimization [13, 14].

The present investigation focused on systematically optimizing abamectin production by *S. avermitilis* using response surface methodology to optimize medium composition and environmental conditions, thereby enhancing abamectin yield. It also evaluated nematicidal activity against *M. incognita* and conducted a preliminary biosafety assessment by estimating its effects on soil microorganisms and human fibroblast cells. This integrated approach provides a robust framework for developing sustainable, safe, and scalable abamectin production strategies for agricultural applications.

## Materials and methods

### *Streptomyces avermitilis* resources

*S. avermitilis* with accession number (OP108264.1) was kindly obtained from the Microbial Inoculants Center (MIC), Faculty of Agriculture, Ain Shams University, Egypt. It was previously isolated by Radwan et al. [1]. The culture was maintained at 4 °C on yeast extract malt extract-glucose (YMG) agar slants [15].

### Root-knot nematode resource

A pure culture of *M. incognita* was reared on Tomato seeds (*Lycopersicon esculentum* Mill., variety 023, susceptible to *M. incognita*), obtained from SAKATA company, Thailand, in a screen house at 30 ± 5 °C using a single egg mass. Newly hatched second-stage juveniles (J_2_s) served as inoculum. The root-knot nematode *M. incognita* was identified from adult female nematodes based on the morphological characteristics of their perineal pattern [16].

### Antimicrobial activities of *S. avermitilis* against bacterial and fungal pathogens

According to Siddique et al. [17], the *S. avermitilis* strain was grown on production medium [15] (Table S1) incubated for 10 days at 28 °C at 150 rpm, and then centrifuged at 8000 rpm for 30 min at 4 °C. The supernatant and the methanol-extract of the mycelium prepared as described by Radwan et al. [1], and were tested for their antimicrobial activities using well diffusion method against two plant bacterial pathogens (*Erwinia carotovora* DSM30170 and *Xanthomonas campestris* ATCC 13951) and six beneficial bacteria (*Serratia marcescens* EMCCN 1068, *Micrococcus luteus* ATCC 9341, *Pseudomonas fluorescens* ATCC 13525, *Bacillus megaterium* ATCC 10778, *Bacillus subtilis* ATCC 35854, and *Bacillus circulans* ATCC 4513) in the presence of Gentamicin (20 µg/ml), Streptomycin (50 µg/ml) as positive control, and five plant fungal pathogens (*Fusarium oxysporum* DSM 20542, *Rhizopus nigricans* DSM 11226, *Aspergillus niger* ATCC10404, *Candida albicans* ATCC 60193 and *Alternaria solani* ATCC58177) in the presence of Nystatin (20 µg/ml), and Metronidazole (50 µg/ml) as positive control using Muller-Hinton agar medium [18] according to the method outlined byHumphries et al. [19]. All the strains were kindly obtained from the Microbial Inoculants Center (MIC) in the Faculty of Agriculture at Ain Shams University in Egypt. The diameter of the inhibition zones was measured in “mm” at the end of the incubation period (2–5 days).

### The cytotoxicity of *S. avermitilis’* mycelial methanol extract

Cytotoxicity of the *S. avermitilis* was conducted according to the method of Elnady et al. [20]. Human skin fibroblast (HSF) cells were obtained from Nawah Scientific Inc., Cairo, Egypt. The cells were maintained at 37 °C in Dulbecco’s Minimum Essential Medium (DMEM) supplemented with 100 mg/mL of streptomycin, 100 units/mL of penicillin, and 10% heat-inactivated fetal bovine serum in a humidified atmosphere of 5% (v/v) CO_2_. Cell viability was assessed using a Sulforhodamine B)SRB(cytotoxicity assay kit as described by Skehan et al. [21]. Aliquots of 100 µL of fibroblast cells suspension (5 × 10^3^ cells/mL) were placed in each well of the 96‐well plates and incubated for 24 h. at 37 °C. HSF cells were treated with 100 µL of mycelium methanol extract (containing the nematicidal components {abamectin}) at gradual concentrations from 20% to 100%. After 72 h. of exposure, cells were fixed by adding 150 µL of 10% Trichloroacetic acid (TCA) followed by incubation at 4 °C for 1 h. The TCA solution was removed, and cells were washed five times with distilled water. Aliquots of 70 µL SRB solution (0.4% w/v) were added, and the plates were incubated in the dark at room temperature for 10 min. The plates were washed three times with (1% v/v) acetic acid and allowed to air‐dry overnight. Then, 150 µL of Tris buffer (hydroxymethyl) aminomethane (10 mM) was added to dissolve the protein‐bound SRB stain. The absorbance was measured at 540 nm using a BMG LABTECH^®^‐FLUO star Omega microplate reader (Ortenberg, Germany). Cell viability was represented as the percentage of control cell viability, which was set as 100%. The percentage of relative viability was calculated using the following equation [22]:

$${\%}\:\mathrm{C}\mathrm{e}\mathrm{l}\mathrm{l}\:\mathrm{v}\mathrm{i}\mathrm{a}\mathrm{b}\mathrm{i}\mathrm{l}\mathrm{i}\mathrm{t}\mathrm{y}=\frac{\mathrm{A}\mathrm{b}\mathrm{s}\mathrm{o}\mathrm{r}\mathrm{b}\mathrm{a}\mathrm{n}\mathrm{c}\mathrm{e}\:\mathrm{o}\mathrm{f}\:\mathrm{t}\mathrm{r}\mathrm{e}\mathrm{a}\mathrm{t}\mathrm{e}\mathrm{d}\:\mathrm{c}\mathrm{e}\mathrm{l}\mathrm{l}\mathrm{s}}{\mathrm{A}\mathrm{b}\mathrm{s}\mathrm{o}\mathrm{r}\mathrm{b}\mathrm{a}\mathrm{n}\mathrm{c}\mathrm{e}\:\mathrm{o}\mathrm{f}\:\mathrm{c}\mathrm{o}\mathrm{n}\mathrm{t}\mathrm{r}\mathrm{o}\mathrm{l}\:\mathrm{c}\mathrm{e}\mathrm{l}\mathrm{l}\mathrm{s}}\:\times\:\:100$$

### Influence of incubation temperatures, pH, and salinity levels on the growth of *S. avermitilis* and its nematicidal potential

The influence of incubation temperatures, pH levels, and salinity on *S. avermitilis* growth and nematicidal potential was estimated using inorganic salt starch liquid medium [23]. Each experiment was conducted by using 5 mL of the strain’s vegetative mycelium to inoculate 50 mL of the liquid medium. In all experiments, the treatments were conducted in triplicate and incubated for 8 days, after which the culture suspension was collected. To examine the effect of incubation temperatures, the inoculated medium was incubated at different temperatures ranging from 15 °C to 40 °C, with 5 °C increments incubated for 8 days at 150 rpm.

To investigate the effect of pH levels, the liquid medium was adjusted to various pH levels ranging from 3 to 11 ± 0.5 using 1 N HCl or 1 N NaOH. The medium was then incubated at 28 °C incubated for 8 days at 150 rpm. The final pH value of the culture medium was recorded at the end of the incubation period.

To examine the effect of salinity, a liquid medium with different NaCl concentrations, ranging from 0.1% to 10% NaCl, was used and incubated at 28 °C incubated for 8 days at 150 rpm. At the end of incubation, the Electrical conductivity (EC) of the medium was measured using an EC meter (Portable High Range EC/TDS Meter - HI99301-Hanna Instruments- USA).

At the end of each previous experiment, the culture suspension was split into two parts. One part was centrifuged at 8000 rpm for 30 min at 4 °C to collect the mycelia-containing pellets, then washed three times with distilled water. These pellets were then dried at 67 °C until a constant weight was reached to determine the mycelial dry weight (g/L). The second part (1 mL) was used for mycelial extraction to evaluate its nematicidal potential as described by Radwan et al. [1]. The nematicidal potential of the extracts derived from *S. avermitilis* was examined on *M. incognita* (J_2_s) [24] as follows: 1 mL of distilled water containing 200 J_2_s/mL was added to 1 mL mycelial methanol extract. The control treatment was prepared by adding 1 mL of the medium solution without bacterial strain to 1 mL of each nematode suspension containing the same number of nematodes. The number of juveniles, both alive and dead, was counted for each treatment using a light microscope after 24 h of the incubation period. Each treatment was prepared with three replicates. The nematodes’ mortality was assessed by observing their straight shape and lack of movement after post-stimulation with a fine needle [25]. The mortality rate of nematodes was calculated as the following equation [26]:

$${\%}\mathrm{M}{o}\mathrm{r}\mathrm{t}\mathrm{a}\mathrm{l}\mathrm{i}\mathrm{t}\mathrm{y}=\frac{\mathrm{C}1\:-\:\mathrm{C}2}{\mathrm{C}1}\times\:100$$

Where: C1 is the number of alive nematode larvae in the control.

 C2 is the number of alive nematode larvae in the treatments.

### Effect of incubation period and growth media on the growth pattern of *S. avermitilis* and the production of nematicidal components

Five mL of the vegetative mycelium inoculum was used to inoculate 50 mL of starch nitrate medium [27] (Table S2) and production medium [15]. The inoculated media were incubated at 28 °C for 21 days at 150 rpm. The culture suspension was collected every 3 days and divided into two portions. The first part was used to estimate the mycelial dry weight (g/L) as mentioned above. The second part (1 mL) was used for mycelial extraction to assess its nematicidal components as described by Radwan et al. [1]. The filtered mycelial extraction were then used to determine the concentration of nematicidal components by HPLC according to the method described by Deng et al. [28]. The amount of consumed starch (carbon source) from the media was estimated using the method described by Sudharhsan et al. [29]. During the logarithmic phase, the specific growth rate (µ), specific abamectin production rate (qP), doubling time (td), multiplication rate (MR), effective yield, mycelium yield efficiency and productivity of nematicidal components (abamectin productivity), were calculated using the following equations [30]:

1. The specific growth rate (d^-1^):

$$\:{\upmu\:}=\frac{\mathrm{l}\mathrm{n}\:\mathrm{x}\:-\:\mathrm{l}\mathrm{n}\:\mathrm{x}0}{\mathrm{t}\:-\:\mathrm{t}0}$$

Where: µ = specific growth rate (d^− 1^), X = Amount of growth after t time (t), and X_0_ = Amount of growth at the beginning time (t_0_).

2. The specific abamectin production rate:

$$\:\mathrm{q}\mathrm{P}=\frac{1}{\mathrm{X}}\times\:\frac{\mathrm{d}\mathrm{P}}{\mathrm{d}\mathrm{t}}$$

Where: P is product concentration and dP/dt is the change in product concentration over time.

3. Doubling time (d):

$$\:\mathrm{t}\mathrm{d}=\frac{\mathrm{l}\mathrm{n}\:2}{{\upmu\:}}$$

4. Multiplication rate:

$$\:\mathrm{M}\mathrm{R}=\frac{1}{\mathrm{t}\mathrm{d}}$$

5. Abamectin yield coefficient relative to biomass (g/g):

$$\:\mathrm{Y}\mathrm{P/X}=\frac{\mathrm{A}\mathrm{m}\mathrm{o}\mathrm{u}\mathrm{n}\mathrm{t}\:\mathrm{o}\mathrm{f}\:\mathrm{a}\mathrm{b}\mathrm{a}\mathrm{m}\mathrm{e}\mathrm{c}\mathrm{t}\mathrm{i}\mathrm{n}\:\mathrm{p}\mathrm{r}\mathrm{o}\mathrm{d}\mathrm{u}\mathrm{c}\mathrm{e}\mathrm{d}\:(\mathrm{g}/\mathrm{L})}{\mathrm{a}\mathrm{m}\mathrm{o}\mathrm{u}\mathrm{n}\mathrm{t}\:\mathrm{o}\mathrm{f}\:\mathrm{b}\mathrm{i}\mathrm{o}\mathrm{m}\mathrm{a}\mathrm{s}\mathrm{s}\:(\mathrm{g}/\mathrm{L})}$$

6. Mcelium yield efficiency

$$\%  = \frac{{{\text{Mycelium\ dry\ weight}}\left( {{\mathrm{g}}/{\mathrm{L}}} \right)}}{{{\text{Initial\ sugar}}\left( {{\mathrm{g}}/{\mathrm{L}}} \right)}}\times 100$$

7. Abamectin productivity (g/L/d)

$$ = \frac{{{\text{Concentration\ of\ Nematicidal\ components}}\left( {{\mathrm{g}}/{\mathrm{L}}} \right)}}{{{\mathrm{Time}}\left( {{\mathrm{days}}} \right)}}$$

### Statistical optimization of abamectin synthesis and mycelium production using response surface methodology (RSM)

The response surface methodology was carried out in two steps. The first step used Taguchi design to screen some nutritional and physical factors to identify the most significant variables for abamectin synthesis and mycelium production. The second step employed the Box-Behnken design to optimize these selected variables for maximum abamectin and mycelium production.

#### Screening of nutritional and physical factors affecting abamectin synthesis and mycelium production using Taguchi design

The Taguchi design was conducted using Design-Expert statistical software (Version 12, Stat-Ease, Inc., Minneapolis, MN, USA) to perform this experiment, as recommended by Osman et al. [31].

A total of seven (n) variables, including four nutritional factors (carbon source, nitrogen source, α-amylase, and CaCO_3_ concentrations) and three physical factors (inoculum size, agitation speed, and incubation period), were studied in 8 (*n* + 1) experiments. Each row represented a trial run, and each column represented an independent variable, as shown in Table (S3). The average of the data was used as the response of the design, and each variable was tested at two levels, high and low, indicated by (+) and (-) signs, respectively (Table 1).

**Table 1:** The Taguchi design’s variables for identifying the significant factors affecting abamectin synthesis and mycelium production by *S. avermitilis*

Symbol	Factors	Level	
		(Low) -1	(High) + 1
A	Carbon source conc. (potato starch) (g/L)	100	140
B	Nitrogen source conc. (Yeast extract) (g/L)	8	16
C	α-amylase (g/L)	0.2	0.5
D	CaCO_3_ (g/L)	0.8	6.0
E	Agitation speed (rpm)	150	200
F	Inoculum size (%)	5	10
G	Incubation period (day)	10	12

#### Maximization of abamectin and mycelium production within *S. avermitilis* using Box-Behnken design

Following the Taguchi design that identified the most significant factors for abamectin synthesis and mycelium production by the *S. avermitilis*, a Box-Behnken design (BBD) was used to optimize these significant variables (carbon source concentration, α-amylase, and incubation period). These variables were examined at three levels (-1, 0, and + 1) (Table 2) and five central points, resulting in 17 batch experiments (Table S3). All variables were taken at a centrally coded value of zero. The statistical Design-Expert software (Version 12, Stat-Ease, Inc., Minneapolis, MN, USA), was used to analyze the experimental data. The culture suspension was collected at the end of the incubation period. The mycelium dry weight (g/L) was estimated, and the abamectin was extracted from the mycelium using the methanol-extract method and quantified by HPLC as described in [1].

**Table 2:** The Box-Behnken design’s variable levels for maximizing abamectin synthesis and mycelium production by *S. avermitilis*

Symbol	Variables	Levels		
		+ 1	0	-1
A	Carbon source conc. (potato starch) (g/L)	140	150	160
B	α-amylase (g/L)	0.1	0.15	0.2
C	Incubation period (day)	8	10	12

### Statistical analysis

Data were analyzed using GraphPad Prism 10 software (Dotmatics, San Diego, USA) and are presented as the mean ± 95% confidence interval (CI), with 5 biological replicates for physiological variables. Significant differences among samples are indicated by different letters.

## Results

### Antimicrobial activities of *S. avermitilis* against bacterial and fungal pathogens

The cell-free supernatant and cell methanol extract showed no antimicrobial activities towards any of the tested pathogens and beneficial microorganisms, as no inhibitory zones were observed (Table 3 and Fig. S1). Therefore, the non-toxicity of *S. avermitilis* towards the tested microorganisms can be authorized.

**Table 3:** Antimicrobial activities of *S. avermitilis* against some common bacteria and fungi

Target bacterial and fungal strains		Inhibition zone (mm)			
		Cell-free supernatant	Methanol extracted mycelium	Positive control	
				Gentamicin (20 µg/mL)	Streptomycin (50 µg/mL)
Pathogenic plant bacteria	*Erwinia carotovora* DSM30170	0	0	18	19
	*Xanthomonas campestris* ATCC 13,951	0	0	17	14
Nonpathogenic plant bacteria	*Serratia marcescens* EMCCN 1068	0	0	15	19
	*Micrococcus luteus* ATCC 9341	0	0	12	15
	*Pseudomonas fluorescens* ATCC 13,525	0	0	23	27
	*Bacillus megaterium* ATCC 10,778	0	0	20	22
	*Bacillus subtilis* ATCC 35,854	0	0	10	15
	*Bacillus circulans* ATCC 4513	0	0	12	15
				Nystatin (20 µg/mL)	Metronidazole (50 µg/mL)
Pathogenic plant fungal	*Fusarium oxysporum* DSM 20,542	0	0	20	13
	*Rhizopus nigricans* DSM 11,226	0	0	22	16
	*Aspergillus niger* ATCC10404	0	0	18	14
	*Candida albicans* ATCC 60,193	0	0	15	10
	*Alternaria solani* ATCC58177	0	0	21	12

### Cytotoxicity of the *S. avermitilis’* mycelial methanol extract

**Fig. 1:**
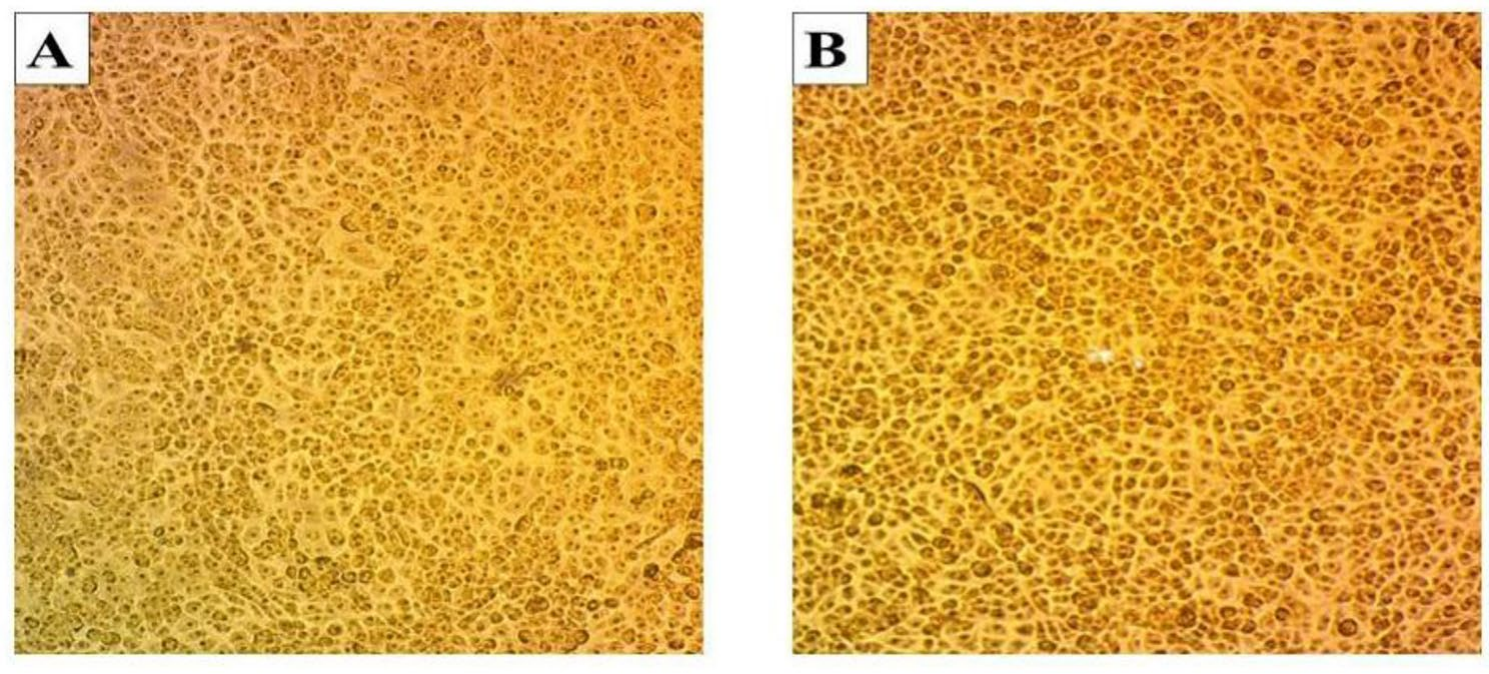
The cytotoxicity of *S. avermitilis*’ mycelial methanol extract on normal HSF cells (**A**) Control treatment shows normal adherent cells. (**B**) The cytotoxicity of *S. avermitilis*’ mycelial methanol extract (100% concentration) results in 90.3% viability with slightly damaged cells, indicated by a reduction in cell adhesion

The mycelial methanol extract of *S. avermitilis* was tested for its cytotoxicity against human skin fibroblast (HSF) cell lines at a range of concentrations from 20%, which contains 64.76 µg/100 µL abamectin, to 100%, which has 323.8 µg/100 µL abamectin. According to the results of Table 4 and Fig. 1, HSF cells showed 98.6% cell viability at 20% mycelial methanol extract. Cell viability decreased as the extract concentration increased, reaching 90.3% at 100% (323.8 µg/100 µL abamectin). HSF cells that received the control treatment exhibited 100% viability. (Table 4; Fig. 1).

The changes in the cell line’s morphology were observed after treatment with various concentrations of mycelial methanol extract using an inverted phase-contrast microscope at 100× magnification (Fig. 1B). HSF cells survived and displayed normal adherence when treated with a mycelial methanol extract of up to 129.52 µg/100 µL. However, at higher concentrations (323.8 µg/100 µL), there was a noticeable change in cell morphology, and a slight decrease in the number of viable cells, indicating cell death compared to the control. Microscopic images showed a slight increase in cytotoxicity of *S. avermitilis* compared to the control treatment (Fig. 1A).

**Table 4:** The cytotoxicity of different concentrations of *S. avermitilis*’ mycelial methanol extract on normal HSF cells

Mycelial methanol extract concentration (%)	Concentration of abamectin in the mycelial methanol extract (µg/100µL)	HSF viability (%)
Control	0.00	100.0 ± 0.00
20	64.76	98.6 ± 0.10
40	129.52	98.2 ± 0.10
60	194.28	97.5 ± 0.12
80	259.04	94.5 ± 0.10
100	323.80	90.3 ± 0.10

*Values are means of 3 replicates; ± Standard deviation (SD)

### The growth performance and nematicidal potentiality of *S. avermitilis* as influenced by incubation temperatures, medium pH and salinity

**Fig. 2:**
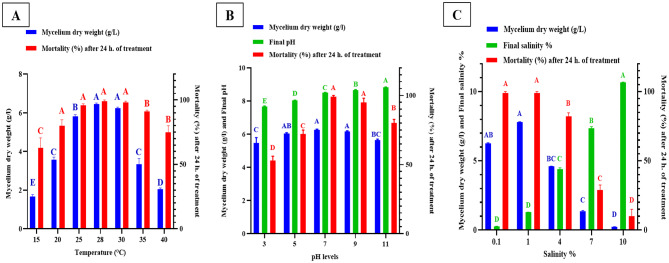
(**A**) Effect of incubation temperature, (**B**) pH, and (**C**) NaCl concentration (%) on the mycelium dry weight and nematicidal potential of *S. avermitilis*’ mycelial extract, grown in inorganic salt starch broth medium at 28℃ for 8 days. *Bar above the column indicates the standard error

The data illustrated in Fig. (2 A) show that the optimal temperature for both *S. avermitilis* growth and nematicidal potential falls between 25 °C and 30 °C. The highest mycelium dry weight of 6.45 g/L and nematicidal potential of 99% were recorded at 28 °C.

The data shown in Fig. (2B) demonstrate that the optimal pH for *S. avermitilis’* growth ranges between pH 5 and 9, while the nematicidal potential was most pronounced between pH 7 and 9. The highest mycelium dry weight of 6.45 g/L and nematicidal potential of 99% were observed at pH 7 after 8 days of incubation at 28 °C. The final pH of the medium at the end of the 8-day incubation period indicated that *S. avermitilis* was able to maintain the pH of the medium within the range of 7.67 to 8.83 across all tested pH levels.

*S. avermitilis* exhibited a noticeable decrease in the growth and nematicidal potential with increasing NaCl concentrations as illustrated in Fig. (2 C). The highest mycelium dry weight and nematicidal potential, of 7.81 g/L and 99% respectively, were recorded at 1% and 0.1% NaCl. The tested strain recorded a slight increase in the final salinity of the medium’s supernatant at the end of the experiment (8-day incubation period) compared to the initial salinity. The increased salinity at the end of the incubation period can be attributed to the production of various metabolites by the tested strain and the reduction in culture volume due to medium evaporation.

### The growth pattern of *S. avermitilis* and abamectin production as affected by the growth media and incubation period

**Fig. 3:**
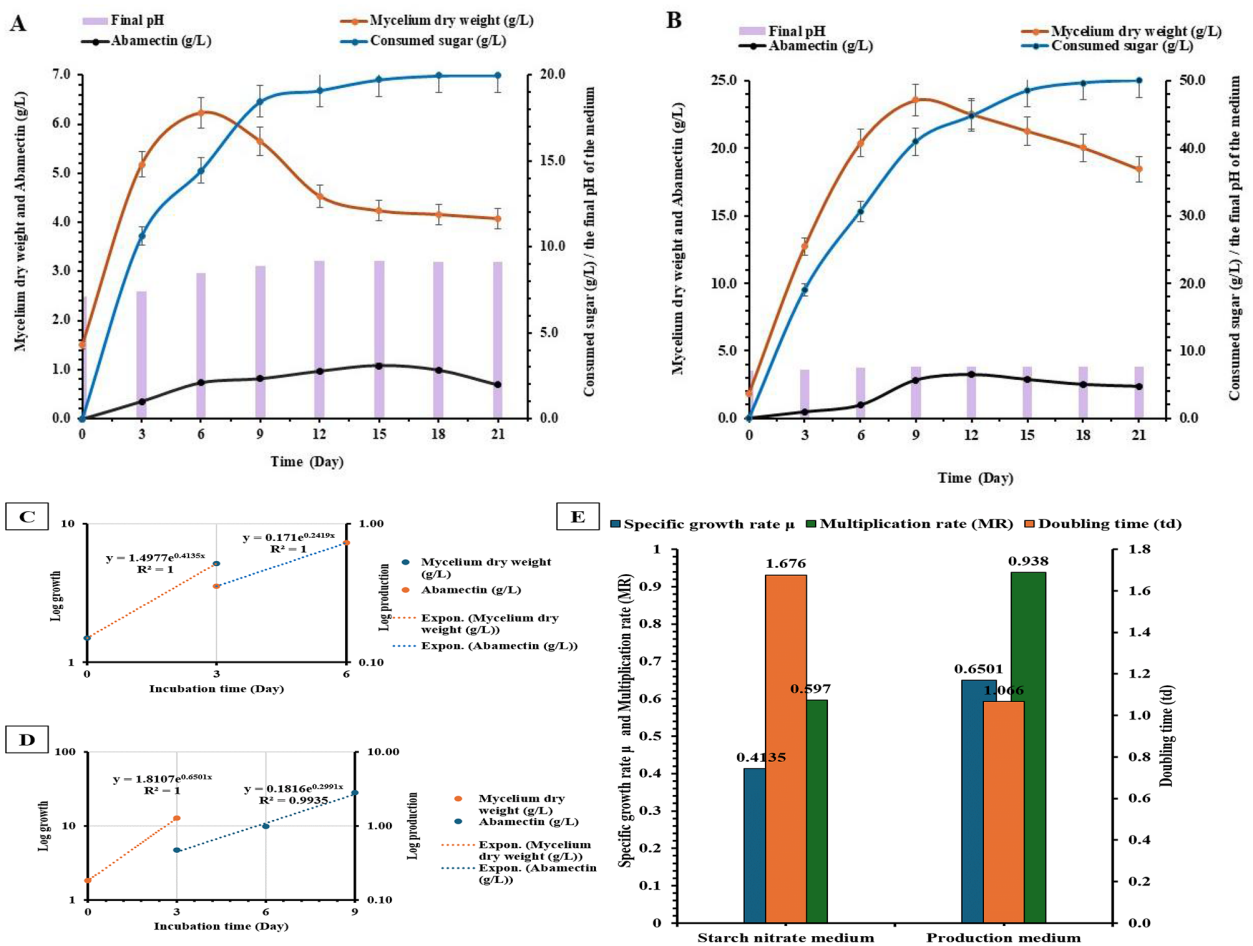
Effect of the incubation period on the growth of *S. avermitilis*, abamectin production, and sugar consumption during a 21-day incubation period at 150 rpm and 28 °C, in: (**A**) starch nitrate medium, and (**B**) production medium, (**C**) the exponential phase of *S. avermitilis* growth and abamectin production in starch nitrate medium, (**D**) the exponential phase of *S. avermitilis* growth and abamectin production in production medium, (**E**) the growth parameters of *S. avermitilis* in starch nitrate and production media

Figures (3 A and B) illustrate the mycelium’s growth pattern (g/L) and abamectin production (g/L) in two growth media over a 21-day incubation period. The lag phase was not observed in both media after the first 3 days. This absence may be attributed to the lengthy interval between the starting point and the first sampling. Therefore, it can be concluded that the lag phase lasts less than three days in both media. Exponential mycelium growth was observed in the starch nitrate broth medium during the first 3 days and in the production medium during the first 6 days, after which growth slowed as it transitioned to the pre-stationary phase. The highest mycelium biomass was 6.23 g/L after 6 days in the starch nitrate medium and 23.57 g/L after 9 days in the production medium, indicating an around 3.78-fold increase in maximum mycelium dry weight in the production medium compared to the starch medium. During the exponential growth of *S. avermitilis*, the mycelium dry weight exhibited a high determination coefficient (R^2^) of 1 across both media (Fig. 3C and D).

Additionally, Fig. (3 A and B) shows low abamectin production during the first three days of fermentation in both starch nitrate and production media. The graph demonstrates that abamectin content rose as the fermentation progressed, and it reached its exponential production from day 3 to day 9 in the production medium and from day 3 to day 6 in the starch nitrate media. This rise occurred around the end of the log and the start of the pre-stationary phases in both media. The highest production levels of 1.07 g/L and 3.23 g/L were achieved after 15 and 12 days of fermentation in the starch nitrate and production media, respectively. Subsequently, abamectin production started to decline, indicating a non-growth-associated process, which is a characteristic of secondary metabolites production (Fig. 3C and D). The calculated growth parameters indicate that the highest µ (0.65/d) and MR (0.93) and the lowest t_d_ (1.06/d) were observed in the production medium. These results demonstrate the superior growth of *S. avermitilis* and abamectin production, which was approximately three times higher in a shorter time, with the highest specific abamectin production rate (qP) of 0.29/d in the production medium Fig. (3E).

The residual sugar content (g/L) was measured, and the amount of sugar consumed was calculated during the fermentation process. The soluble corn starch, which acts as the carbon source, was fully depleted during the growth of *S. avermitilis* and abamectin production by the end of fermentation in both media as presented in Figs. (3 A and B).

After 21 days of incubation, the pH of the starch medium increased to 9.1, while the production medium remained close to neutral (pH 7.6). The correlation between mycelium growth, abamectin production, and sugar consumption was analyzed (Table 5). Day 9 revealed the highest abamectin productivity (0.31 g/L/d) and mycelium yield efficiency (47.14%) in the production medium, which correlated with the lowest abamectin yield coefficient relative to mycelium biomass (Yp/x) of 0.12 g/g. These results suggest that the production medium is more optimal for growth and the synthesis of abamectin than the starch medium (Table 5).

**Table 5:** The correlation between the growth of *S. avermitilis*, abamectin production, and sugar consumption in starch nitrate and production media incubated at 28 °C for 21 days at 150 rpm

Time (days)	Starch nitrate medium			Production medium		
	Abamectin productivity (g/L/d)	Mycelium yield efficiency (%)	Abamectin yield coefficient relative to mycelium biomass (g/g)	Abamectin productivity (g/L/d)	Mycelium yield efficiency (%)	Abamectin yield coefficient relative to mycelium biomass (g/g)
0	0.00	7.50	0.00	0.00	3.62	0.00
3	0.11	25.90	0.06	0.15	25.46	0.03
6	0.12	31.15	0.11	0.16	40.80	0.04
9	0.09	28.25	0.14	0.31	47.14	0.12
12	0.08	22.65	0.21	0.26	45.04	0.14
15	0.07	21.15	0.25	0.19	42.56	0.13
18	0.05	20.80	0.23	0.13	40.08	0.12
21	0.03	20.40	0.16	0.11	36.90	0.12

### Statistical optimization of abamectin synthesis and mycelium production using response surface methodology design (RSM)

#### Screening of nutritional and physical factors affecting abamectin synthesis and mycelium production by *S. avermitilis* using Taguchi design

Taguchi design was used to identify the main nutritional and physical factors that affect the growth of *S. avermitilis* and the production of abamectin. Data presented in Table 6 show that highest mycelium dry weight of 60.65 g/L and abamectin production of 7.09 g/L were observed in run no. 4. In this run, the factors potato starch, yeast extract, agitation speed, and inoculum size were at a high level (+ 1), while the factors α-amylase, CaCO_3_ and incubation period, were at a low level (-1). Run No. 8 recorded the lowest mycelium dry weight and abamectin production with 36.10 g/L and 3.95 g/L, respectively. In this run, the parameters potato starch, inoculum size, and incubation period were at a high level, while the other parameters were at a low level.

The following two equations were generated by the software to calculate the mycelium dry weight (g/L) and abamectin production (g/L):

$$\eqalign{ {{\rm{Y}}_{{\rm{Mycelium}}\,{\rm{dry}}\,{\rm{weight}}}}{\rm{ = }} & {\rm{ + 50}}{\rm{.21 + 6}}{\rm{.82}}\left( {{\rm{Potato}}\,{\rm{starch}}\,{\rm{conc}}{\rm{.}}} \right) \cr & {\rm{-- 0}}{\rm{.70 }}\left( {{\rm{Yeast}}\,{\rm{extract}}\,{\rm{conc}}{\rm{.}}} \right) \cr & {\rm{--0}}{\rm{.28 }}\left( {{\rm{\alpha - amylase}}\,{\rm{conc}}{\rm{.}}} \right) \cr & {\rm{--3}}{\rm{.97 }}\left( {{\rm{CaC}}{{\rm{O}}_{\rm{3}}}{\rm{conc}}{\rm{.}}} \right) \cr & {\rm{--2}}{\rm{.51 }}\left( {{\rm{Inoculum}}\,{\rm{size}}} \right) \cr & {\rm{--1}}{\rm{.03 }}\left( {{\rm{Agitation}}\,{\rm{speed}}} \right) \cr & {\rm{ + 1}}{\rm{.06 }}\left( {{\rm{Incubation}}\,{\rm{period}}} \right){\rm{.}} \cr} $$

$$\eqalign{ {{\rm{Y}}_{{\rm{Abamectin}}}}{\rm{ = }} & {\rm{ + 5}}{\rm{.43 + 1}}{\rm{.11}}\left( {{\rm{Potato starch conc}}{\rm{.}}} \right) \cr & {\rm{-0}}{\rm{.43}}\left( {{\rm{Yeast extract conc}}{\rm{.}}} \right) \cr & {\rm{-0}}{\rm{.17}}\left( {{\rm{\alpha - amylase conc}}{\rm{.}}} \right) \cr & {\rm{-0}}{\rm{.30}}\left( {{\rm{CaC}}{{\rm{O}}_{\rm{3}}}{\rm{conc}}{\rm{.}}} \right) \cr & {\rm{-0}}{\rm{.02}}\left( {{\rm{Inoculum size}}} \right) \cr & {\rm{ + 0}}{\rm{.03}}\left( {{\rm{Agitation speed}}} \right) \cr & {\rm{-0}}{\rm{.52}}\left( {{\rm{Incubation period}}} \right){\rm{.}} \cr} $$

The results obtained were statistically analyzed following ANOVA to determine the significance of the variables and any possible interaction (Table 6). The significant of variables was identified based on a high F-value and a low *p-*value (*p* ≤ 0.05) (models with *p-*values below 0.05 were considered significant). Among the seven independent variables, potato starch, α-amylase, and incubation period - were found to significantly impact mycelium dry weight and abamectin production. The remaining factors were not found to be significant (*p ≥* 0.05).

**Fig. 4:**
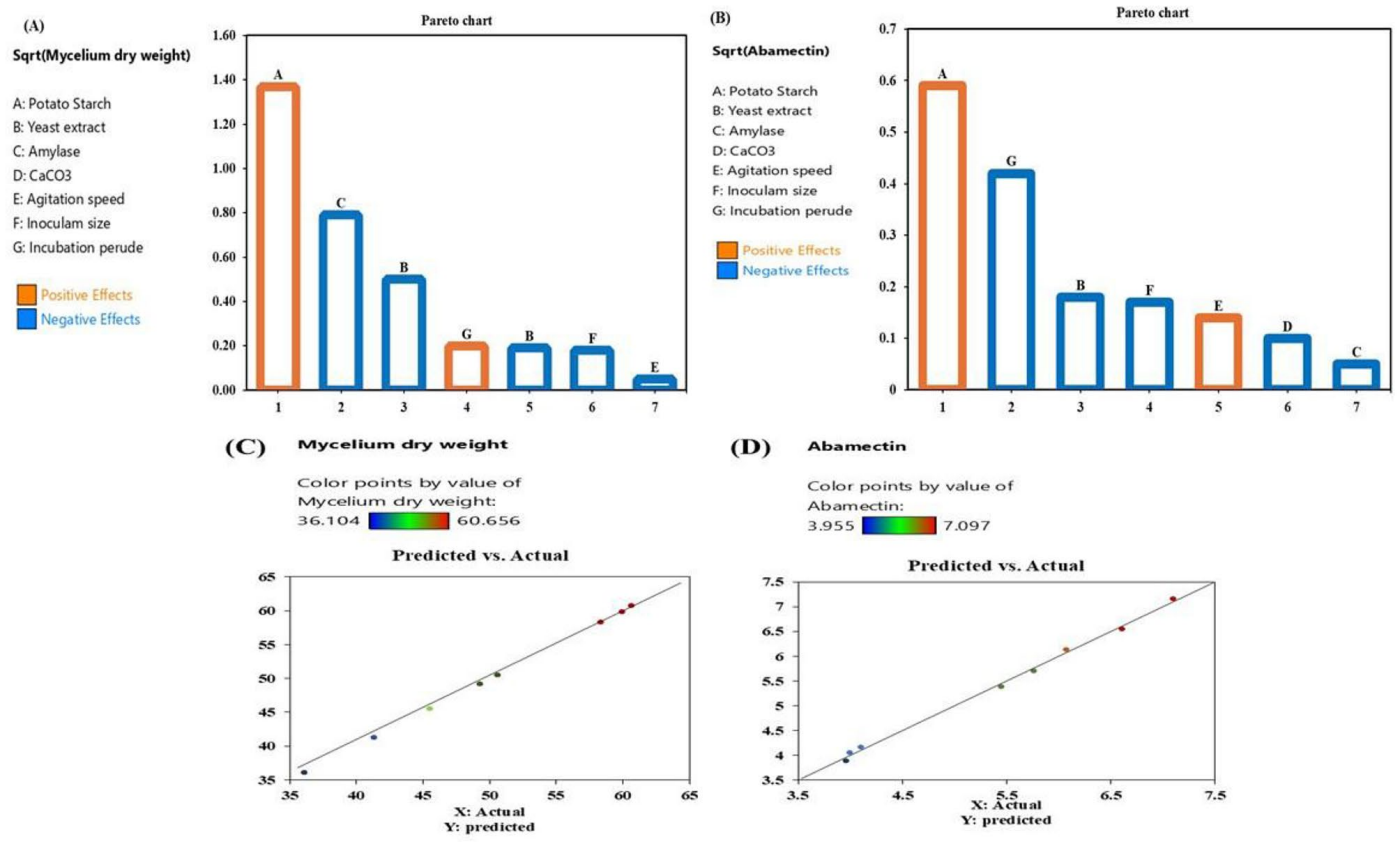
Pareto chart illustrating the contribution effects of different variables on: (**A**) mycelium dry weight and (**B**) abamectin production by *S. avermitilis*, based on Taguchi design observations (the orange color indicates positive effects, and the blue color indicates negative effects). The correlation of predicted and actual values for (**C**) mycelium dry weight and (**D**) abamectin production by *S. avermitilis* using Taguchi design

The determination coefficient (R²) of 0.99 indicates a strong correlation among all factors, levels, and responses, as well as between the experimental and predicted values, demonstrating that the model explains the total variation and the significant effects of these factors on abamectin synthesis and mycelium production (Table 6; Fig. 4A and B). A regression model with an R² value greater than 0.9 is considered to have a very high correlation, confirming a good fit between the predicted and observed values (Fig. 4C and D). These results are illustrated in Fig. (4 A and B), which indicate that potato starch and incubation period positively affected mycelium dry weight and abamectin production (orange columns), while other factors had negative effects (blue columns). Additionally, Fig. (4) demonstrates the influence of all tested factors on mycelium and abamectin production by *S. avermitilis*, revealing a strong correlation between predicted and actual values of mycelium growth and abamectin production, thus confirming the model’s suitability for the study.

The significant factors were selected for the next optimization step using Box-Behnken design (BBD) to determine the optimal level for each significant factor for mycelium and abamectin production.

**Table 6:** The effect of seven independent factors with two factorial levels on mycelial dry weight and abamectin production by *S. avermitilis* using Taguchi design

Run no.	Variables							Responses	
	A	B	C	D	E	F	G	Mycelium dry weight (g/L)	Abamectin contents (g/L)
1	+1	-1	+1	-1	+1	-1	+1	58.31	6.61
2	-1	+1	+1	-1	-1	+1	+1	41.34	3.99
3	+1	+1	-1	+1	-1	-1	+1	59.94	5.45
4	+1	+1	-1	-1	+1	+1	-1	60.65	7.09
5	-1	-1	-1	-1	-1	-1	-1	50.60	5.76
6	-1	-1	-1	+1	+1	+1	+1	45.51	4.10
7	+1	-1	+1	+1	-1	+1	-1	49.23	6.07
8	-1	+1	+1	+1	+1	-1	-1	36.10	3.95

Statistical analysis of variation (ANOVA)									
Variables				Mycelium dry weight (g/L)				Abamectin contents (g/L)	
				*F*-Value		*p*-value (Prob>F)		*F*-Value	*p*-value (Prob>F)
Model				2627.46		0.014*		1262.18	0.02*
A- Potato starch (g/L)				10297.35		0.006*		5119.17	0.01*
B- Yeast extract (g/L)				109.04		0.06		798.52	0.14
C- α-amylase (g/L)				3476.61		0.01*		132.52	0.03*
D- CaCO_3_ (g/L)				139.76		0.11		384.03	0.32
E- Agitation speed (rpm)				0.0003		0.98		3.17	0.21
F- Inoculum size (%)				233.56		0.14		1.66	0.41
G- Incubation period (day)				250.44		0.04*		1137.43	0.01*
Std. Dev.				0.19		0.04			
R^2^				0.99		0.99			

*=Significant at 5% level (*p* ≤ 0.05). *p*-value= probability value, Std. Dev= standard division and R^2^= coefficient of determination. Degree of freedom (df) in the model = 7

#### Maximization of abamectin and mycelium production within *S. avermitilis* using Box-Behnken design

Data presented in Table 7 reveal that runs number 2, 3, 10, 12, and 13, with 150 g/L potato starch, 0.15 g/L α-amylase, and a 10-day incubation period, recorded the highest values of mycelium dry weight and abamectin production of 70.81 g/L and 8.30 g/L, respectively. While the run number 8 achieved the highest mycelium dry weight of 71.08 g/L.

The lowest mycelium dry weights of 61.56 and 63.99 g/L, along with abamectin production of 7.02 and 6.81 g/L, were recorded in runs 14 and 4, respectively, which had the lowest levels of potato starch and α-amylase.

The model’s F-values for mycelium dry weight and abamectin production were 491.56 and 822.16, respectively, indicating that the model was highly significant. Furthermore, the p-value was less than 0.0001, suggesting that the model was highly significant and that the regression model adequately explained the observed variations.

The coefficients of the three variables in the model (potato starch concentration (A), α-amylase concentration (B), and incubation period (C)), as well as the interaction between two variables (AB, AC, and BC) and the quadratic of the three variables (A^^2^, B^^2^, and C^^2^), revealed a significant influence on abamectin and mycelium production.

The model’s determination coefficient R² was 0.99 for mycelium and abamectin production, indicating that 99% of the total variation was explained. This strong correlation between experimental results and predicted values demonstrates the model’s reliability and efficiency for mycelium and abamectin production.

**Table 7:** The actual values of mycelium and abamectin production by *S. avermitilis* displayed in the Box-Behnken matrix for three independent variables

Run no.	Variables					Responses			
	Potato starch (g/L)	α-amylase (g/L)			Incubation period (day)	Mycelium dry weight (g/L)			Abamectin (g/L)
1	+ 1	0			-1	70.92			7.20
2	0	0			0	70.81			8.30
3	0	0			0	70.81			8.30
4	-1	0			-1	63.99			6.81
5	0	-1			+ 1	69.56			7.80
6	-1	0			+ 1	65.42			7.18
7	+ 1	0			+ 1	69.56			7.61
8	0	+ 1			+ 1	71.08			7.82
9	+ 1	+ 1			0	68.29			7.52
10	0	0			0	70.81			8.30
11	0	+ 1			-1	70.72			7.35
12	0	0			0	70.81			8.30
13	0	0			0	70.81			8.30
14	-1	-1			0	61.56			7.02
15	0	-1			-1	69.94			7.39
16	-1	+ 1			0	64.21			7.05
17	+ 1	-1			0	67.64			7.52

Statistical analysis of variance (ANOVA)									
Variables			Mycelium dry weight (g/L)				Abamectin (g/L)		
			F-Value	*p*-value (Prob > F)			F-Value	*p*-value (Prob > F)	
Model			491.56	< 0.0001*			822.16	< 0.0001*	
Potato starch (g/L)			1717.88	< 0.0001*			704.46	< 0.0001*	
α-amylase (g/L)			119.36	< 0.0001*			0.13	0.72	
Incubation period (day)			0.01	0.92			600.76	< 0.0001*	
AB			30.49	0.0009*			0.45	0.52	
AC			59.34	0.0001*			0.34	0.57	
BC			4.16	0.08			1.24	0.30	
A^^2^			2179.28	< 0.0001*			3669.19	< 0.0001*	
B^^2^			206.26	< 0.0001*			742.45	< 0.0001*	
C^^2^			78.11	< 0.0001*			1132.95	< 0.0001*	
Std. Dev.			0.18	0.02					
R^2^			0.99	0.99					

*=Significant at 5% level (*p* ≤ 0.05). *p*-value= probability value, Std. Dev= standard division and R^2^= coefficient of determination

The regression equations illustrate the relationships among variables A, B, and C, and their effects on abamectin and mycelium production. The interpretation of data relies on the signs of the coefficients, which reveal positive (synergistic) or negative (antagonistic) effects on the response, along with interactions among the factors.

The final regression equation to produce mycelium and abamectin production were as follow:

$$\eqalign{ {{\rm{Y}}_{{\rm{Mycelium dry weight}}}}{\rm{ = }} & {\rm{ - 962}}{\rm{.06 + 13}}{\rm{.12 (Potato}}\,{\rm{starch)}} \cr & {\rm{ + 297}}{\rm{.59 }}\left( {{\rm{\alpha - amylase}}} \right) \cr & {\rm{ + 1}}{\rm{.05 }}\left( {{\rm{Incubation}}\,{\rm{period}}} \right) \cr & {\rm{- 1}}{\rm{.00 }}\left( {{\rm{Potato starch*\alpha - amylase}}} \right) \cr & {\rm{ + 0}}{\rm{.03}}\left( {{\rm{Potato starch* Incubation}}\,{\rm{period}}} \right) \cr & {\rm{ + 1}}{\rm{.85 }}\left( {{\rm{\alpha - amylase * Incubation}}\,{\rm{period}}} \right) \cr & {\rm{ - 0}}{\rm{.04(Potato}}\,{\rm{starc}}{{\rm{h}}^{\rm{2}}}{\rm{)}} \cr & {\rm{-507}}{\rm{.0 }}\left( {{\rm{\alpha - amylas}}{{\rm{e}}^{\rm{2}}}} \right) \cr & {\rm{- 0}}{\rm{.19 }}\left( {{\rm{Incubation}}\,{\rm{perio}}{{\rm{d}}^{\rm{2}}}} \right) \cr} $$

$$\eqalign{ {{\rm{Y}}_{{\rm{Abamectin}}}}{\rm{ = }} & {\rm{ - 166}}{\rm{.7 + 2}}{\rm{.13 (Potato}}\,{\rm{starch)}} \cr & {\rm{ + 39}}{\rm{.13 }}\left( {{\rm{\alpha - amylase}}} \right) \cr & {\rm{ + 1}}{\rm{.98}}\left( {{\rm{Incubation}}\,{\rm{period}}} \right){\rm{ }} \cr & {\rm{ - 0}}{\rm{.01 }}\left( {{\rm{Potato starch*\alpha - amylase}}} \right) \cr & {\rm{ + 0}}{\rm{.0003 }}\left( {{\rm{Potato starch* Incubation}}\,{\rm{period}}} \right) \cr & {\rm{ + 0}}{\rm{.13 }}\left( {{\rm{\alpha - amylase * Incubation}}\,{\rm{period}}} \right) \cr & {\rm{ - 0}}{\rm{.007(Potato}}\,{\rm{starc}}{{\rm{h}}^{\rm{2}}}{\rm{)}} \cr & {\rm{ - 126}}{\rm{.6 }}\left( {{\rm{\alpha - amylas}}{{\rm{e}}^{\rm{2}}}} \right){\rm{ }} \cr & {\rm{ - 0}}{\rm{.09}}\left( {{\rm{Incubation perio}}{{\rm{d}}^{\rm{2}}}} \right) \cr} $$

**Fig. 5:**
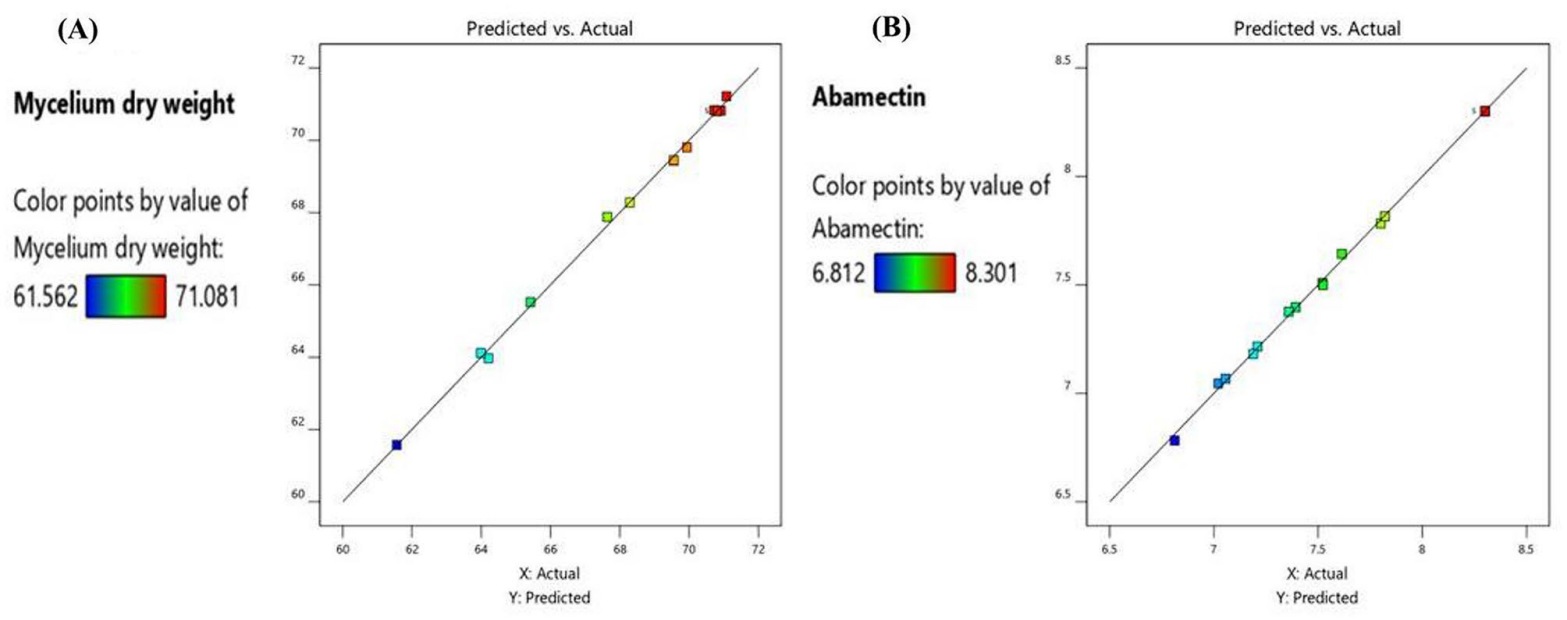
Correlation between predicted and actual values of (**A**) mycelium dry weight and (**B**) abamectin production by *S. avermitilis* using the Box-Behnken design

The correlation between the predicted and actual values is illustrated in Fig. (5), which demonstrates that the actual values were consistent with the predicted values. This alignment suggests that the factors were highly identified within the model.

**Fig. 6:**
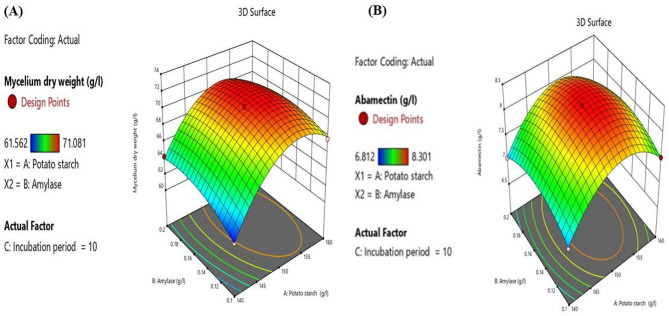
3D contours illustrate the effect of the interaction among all factors on (**A**) mycelium dry weight and (**B**) abamectin production by *S. avermitilis*

The interaction among all factors was demonstrated by three-dimensional contours, which are graphical charts based on the model equation to determine the optimum level of each factor for mycelium dry weight and abamectin production (Fig. 6A and B). Each contour displayed the interaction between two factors while the third factor was held at the zero level. The highest response score occurred when there was a perfect interaction between the independent variables, as indicated by the central point within the highest red contour area, representing the optimal conditions and concentrations of the variables.

According to the study’s findings, the optimal levels of the process variables for maximum production of mycelium and abamectin by *S. avermitilis* can be achieved in a medium consisting of the following: 150 g/L potato starch, 8.0 g/L yeast extract, 0.1 g/L KCl, 0.8 g/L CaCO_3_, 0.1 g/L MgSO_4_, 0.5 g/L NaCl, 0.15 g/L α-amylase, with a pH of 7. The medium should be inoculated with a 10% inoculum size and incubated for 10 days at 28 °C with 200 rpm agitation (Table S5 and Fig S2).

## Discussion

### Safety assessment of *Streptomyces avermitilis* metabolites for biotechnological application

The increasing reliance on biopesticides in modern agriculture necessitates rigorous safety evaluation, particularly regarding their effects on non-target microorganisms and human health [32]. Abamectin, produced by *S. avermitilis*, is widely applied for the control of soil-borne pests; however, its ecological compatibility remains a subject of ongoing investigation [33]. Soil and rhizosphere microorganisms play essential roles in nutrient cycling and plant health, and maintaining their functional stability is critical for sustainable agroecosystems [34].

In the present study, neither the cell-free supernatant nor the mycelial methanol extract exhibited inhibitory effects against the tested plant pathogenic or beneficial rhizosphere-associated microorganisms. This absence of antimicrobial activity suggests that abamectin production under the examined conditions does not adversely affect non-target microbial populations relevant to soil fertility and biofertilization. These findings are consistent with previous reports indicating that avermectins show limited toxicity toward microbial communities, reinforcing their suitability for integration into sustainable biotechnological pest control strategies [35, 36].

Human safety is a critical consideration for any bio-based product intended for widespread application. Using human skin fibroblasts as an in vitro exposure model, the mycelial methanol extract demonstrated low cytotoxicity, particularly at lower concentrations. This result aligns with existing toxicological studies describing abamectin as having relatively low mammalian toxicity compared with many conventional chemical pesticides [37, 38]. While high-dose exposure effects cannot be excluded, the present findings support the classification of abamectin as a comparatively safe biorational compound when produced and applied within controlled limits [39].

### The growth performance and nematicidal potentiality of *S. avermitilis* as influenced by incubation environmental conditions

Secondary metabolite biosynthesis in *Streptomyces* species is tightly regulated by environmental conditions, particularly temperature, pH, and salinity [40]. Abamectin production typically initiates during the transition from exponential growth to the stationary phase, making it especially sensitive to factors that influence cellular metabolism, enzyme activity, and physiological differentiation [41].

Our results showed a decrease in the activity of abamectin over time, which is consistent with Awasthi et al. [42], who stated an increase in the degradation of abamectin at different temperature after 10 days of production, and the highest mycelial growth and anthelmintic activity was occurred between 28 and 30 °C, at which 28 °C represented the optimal mycelial growth and anthelmintic activity. Also, these results align with [40], who stated that *Streptomyces* sp. could be classified as a mesophilic organism. Correspondingly, mycelium growth and anthelmintic activity began to decline at 35 °C, which aligns with previous reports by Siddique and Qureshi [15] and Pan and Cai [43], who explained the reduction of the anthelmintic activity as a result of decreasing the abamectin produced. Therefore, these results could be attributed to the reduction in mycelium growth and secondary metabolite production due to the impact of temperature on enzyme production and activity, as well as the physiological processes of the bacterial cells [44]. In the same context [45], reported that abamectin, which is a neurotoxic pesticide, revealed greater toxicity to insects at lower temperatures due to increased stability of sodium channels and their influx compared to higher temperatures, which raises the vulnerability of the nervous system to the toxic effects of this compound in high temperature [46].

The pH of the culture medium is one of the most important environmental factors [47], as it significantly affects the activity of several enzymes that catalyze metabolic reactions and also influences complex physiological phenomena such as membrane permeability, cell morphology [48], and many cellular processes like regulation and biosynthesis of secondary metabolites [49].

Our findings reveal that *S. avermitilis* has a neutrophilic nature, which agrees with those of [50], who reported that the ideal pH for mycelial growth and secondary metabolite production in *Streptomyces* cultures is usually near neutral. Similar results have been observed for several *Streptomyces* species by Singh et al. [51], who noted that the growth of the *Streptomyces* sp. decreased beyond pH values of 6.5 to 7.5. Likewise [52], found that an optimal pH of 7 increased abamectin production and microbial growth, while deviations from this pH reduced both. Also, a study by Siddique and Qureshi [15] reported that maintaining the medium’s pH between 7.0 and 8.5 increased ivermectin production.

In our research, the final pH after 8 days of incubation ranged from 7.67 to 8.83. This pH stability may result from the buffering effect of microbial metabolites and could explain the significant activity of *Streptomyces* observed during the incubation period.

While Awasthi et al. [42]. reported that the stability of Avermectin (AVMs) is greatly affected by acidic and alkaline conditions [53], acidic conditions promote glycolysis, producing monosaccharide and aglycone derivatives [53], whereas alkaline hydrolysis causes epimerization of stereoisomers and regioisomers (2-epi- and D2,3-AVM), and it is known that these hydrolysis products lack anthelmintic properties [54]. Additionally [42], stated that activity loss due to hydrolysis is greater under alkaline conditions than in acidic conditions. Finally, Naveena et al. [44] pointed out that a shift in the medium’s pH can reduce mycelium growth and secondary metabolite production, due to the effects of pH on enzyme activity and bacterial cell physiology.

Salinity exerted a measurable influence on culture performance, with low NaCl concentrations supporting optimal growth and metabolite synthesis [55]. Elevated salinity levels likely impose osmotic stress and ionic imbalance, resulting in reduced metabolic efficiency and secondary metabolite output [56]. Although *S. avermitilis* cannot be classified as halophilic, its moderate salt tolerance may be advantageous for biotechnological applications in variable soil or fermentation environments [57].

### The growth pattern of *S. avermitilis’* abamectin production as affected by the growth media and incubation period

Several experiments were carried out to study mycelial growth and abamectin production, aiming to boost avermectin yield for use as a pesticide and anthelmintic [58]. The production medium was found to be superior in mycelium yield, abamectin yield coefficient relative to biomass, and abamectin productivity. These results align with those reported by Qiu et al. [59], who observed that the growth of *Streptomyces* and abamectin production, as well as the timing of peak production, varied depending on the strain, medium composition, incubation temperature, inoculum size, and medium pH.

According to Siddique et al. [17], avermectin B1b reaches its peak production after 9 to 14 days of fermentation at 28 to 30 °C. Following this, there is a gradual decline, which aligns with our findings that mycelium growth and avermectin production peak between 9 and 12 days of fermentation at 28 °C. In the production medium, we achieved a maximum specific growth rate (µ max) of 0.6501/d, a cell dry weight of 23.57 g/L, and abamectin production of 3.23 g/L, which could be attributed to the higher concentration of soluble corn starch as a carbon source compared to the starch nitrate medium [8, 15].

### Statistical optimization of abamectin synthesis and mycelium production using response surface methodology design (RSM)

Enhancing the production of abamectin for market use as a pesticide and anthelmintic agent is considered a major challenge. The nutritional requirements and physical conditions necessary for the growth of *Streptomyces* sp. play crucial roles during metabolites synthesis [15, 31]. Factors such as carbon source, nitrogen source, α-amylase, CaCO_3_, agitation speed, inoculum size, and incubation duration are essential for bacterial growth and metabolites production in the media [60–62].

*Streptomyces* sp., particularly in secondary metabolite production, favors starch as a carbon source, as reported by several researchers. Al Farraj et al. [63] similarly found that starch enhances avermectin B1a production in *S. avermitilis*. These findings align with our Taguchi design results, highlighting the importance of potato starch for *S. avermitilis* growth and abamectin production, likely due to starch’s slow digestion promoting secondary metabolite synthesis [62].

The source of nitrogen is essential for the growth of microorganisms, as it is a primary component of protein synthesis, which makes up a significant part of all living organisms. It is also crucial for supporting growth and regulating the production of secondary metabolites in *Streptomyces* sp [64]., which is believed to be linked to the gradual release of nitrogenous compounds during fermentation. Several studies have shown that yeast extract encourages rapid growth in *Streptomyces* strains [65]. The results obtained from the Taguchi design indicated that excessive amounts of yeast extract can potentially inhibit abamectin synthesis and mycelium growth. These findings agree with those of Gao et al. [8], who reported decreased mycelium growth and lower secondary metabolite production with excess nitrogen in the medium. Therefore, this study suggests reducing yeast extract appropriately to achieve the best results.

In the present study, inoculum sizes ranging from 5% to 10% were selected for their optimal impact on mycelium growth and avermectin production. The results indicated that both abamectin production and mycelium growth reached their highest levels at an inoculum size of 10% (v/v). These results align with those reported by Osman et al. [31] and Barbuto et al. [67], who stated that a 10% inoculum revealed the highest significant mycelium growth and abamectin production. Furthermore, Siddique et al. [17] stated that increasing the inoculum size by more than 10% could reduce avermectin B1b production by *S. avermitilis* cells due to increased competition among mycelial cells for nutrient requirements.

Although *Streptomyces* strains produce alpha-amylase for starch hydrolysis, various studies have demonstrated that adding external alpha-amylase to the medium can more effectively enhance the conversion of starch to glucose [68]. This mechanism enhances carbon flux without causing early carbon catabolite repression, thereby favoring secondary metabolite accumulation rather than primary growth [31].

The incubation period is a crucial factor that greatly affects both mycelium growth and abamectin production [64]. In this study, abamectin yield increased with longer incubation in the growth medium. The highest production was achieved on the 10th day of fermentation, aligning with previous findings by Siddique et al. [17]. The production peak started to decline, which could be attributed to the depletion of most nutrients, especially soluble starch, after the 10th day [17].

Calcium carbonate (CaCO₃) can maintains pH balance during fermentation by neutralizing acids, producing neutral salts, and releasing carbon dioxide (CO₂) [7]. Selecting CaCO₃ was crucial to assess its impact on mycelium growth and abamectin production. In this study, CaCO₃ had no significant effect on either mycelial growth or abamectin production. However, Chen et al. [12] demonstrated the positive effect of CaCO₃ on microbial growth and antifungal metabolite production by *S. alfalfae* XN-04.

While selecting the right nutrients is crucial in *Streptomycetes* cultures, oxygen regulation is equally important for balancing secondary metabolite production and biomass growth. Therefore, ensuring an adequate oxygen transfer rate in the medium by adjusting agitation speed or aeration flow without stressing the microorganisms in the culture was the goal of this experiment to achieve optimal mycelium growth and abamectin production. Adequate dissolved oxygen levels during fermentation promote optimal mycelial growth and differentiation, which in turn affects secondary metabolite production [66, 67]. Increasing oxygen levels with higher agitation speeds might be counterproductive, as it can damage the mycelium and ultimately decrease secondary metabolite yields [67, 69]. In this regard, Song et al. [70] and Barbuto et al. [67] stated that the production of nematicidal components is significantly influenced by the percentage of dissolved oxygen in the growth medium of *Streptomyces* sp. Conversely, Siddique and Qureshi [15] reported that agitation speed has minimal impact on microbial growth and enzyme production during fermentation. The mycelial growth and abamectin yield in *S. avermitilis* showed minimal variation with changes in agitation speed, with 200 rpm identified as optimal, which aligns with Gao et al. [8].

The value of the determination coefficient (R²) should range from 0 to 1. Values closer to 1 indicate a stronger model with better predictive ability [62, 71, 72, 73]. Both the Taguchi and Box-Behnken designs produced a determination coefficient (R²) of 0.99. This result confirms the significance of the models and aligns with the findings of Selvaraj et al. [62] and Souagui et al. [65].

The significance of the input variables was indicated by higher calculated F values and lower *P* values (less than 0.1) in the ANOVA test [11]. Furthermore, the significance of the models is demonstrated by their *P* values, which are essential for understanding how variables interact. Factors in the models are considered significant if the *P* value is less than 0.05 [62]. In this study, both models showed *P* values below 0.05 (*P <* 0.05), indicating that the model factors are significant (Souagui et al., 2019) [61, 62].

## Conclusion

The present study demonstrated the potential of *S. avermitilis* as a robust natural producer of abamectin with high nematicidal activity against *M. incognita*. Environmental factors such as temperature, pH, and salinity significantly affect mycelial growth and metabolite production, underscoring the importance of controlled fermentation conditions. Optimization of the production medium significantly increased both biomass and metabolite yields, highlighting its relevance to the development process. Statistical optimization identified potato starch, α-amylase concentration, and incubation period as key factors influencing abamectin productivity. The application of response surface methodology was effective for process optimization and provides a practical framework for future pilot- and industrial-scale studies. The *S. avermitilis* extract exhibited no inhibitory effects on beneficial or pathogenic soil microorganisms and showed minimal cytotoxicity toward human fibroblast cells, confirming its biosafety and potential application as an eco-friendly biocontrol agent.

Future studies should focus on the scalability of abamectin production by assessing *S. avermitilis* performance in large-scale fermentation and validating the production process under field conditions.

## Supplementary Information

Below is the link to the electronic supplementary material.

Supplementary Material 1

Supplementary Material 2

## Data Availability

The data sets used and/or analyzed during the current study are available from the corresponding author upon reasonable request. *Streptomyces avermitilis* MICNEMA2022 with accession number (*OP108264.1*) was deposited in GenBank at (https://www.ncbi.nlm.nih.gov/nuccore/OP108264).
