# Supplementary material for: Optimization of abamectin production by Streptomyces avermitilis and its antagonistic activity against Meloidogyne incognita
Source: BMC Biotechnol. 2026 Mar 11;26:41. doi: 10.1186/s12896-026-01112-6 (PMC13064350; doi:10.1186/s12896-026-01112-6)
Supplement: Supplementary file 1 — Supplementary Material 1 [file 12896_2026_1112_MOESM1_ESM.docx]

**Table S1** Starch nitrate medium **(Waksman, 1961)**

| Components | (g/L) |
| --- | --- |
| Starch | 20.0 |
| Potassium nitrate | 2.00 |
| Dipotassium hydrogen phosphate | 1.00 |
| Sodium chloride | 0.50 |
| Magnesium sulphate | 0.50 |
| Calcium carbonate | 3.00 |
| *Trace salts solution **(Pridham *et al.,* 1958)** | 1.00 ml |
| pH | 7.0-7.2 |
| *Trace salts solution as described by **Pridham *et al.* (1958).** It comprises 0.1 g/L of each of the following salts: ferrous sulphate, magnesium chloride, copper sulphate, and zinc sulphate. | |

**Table S2** Production medium **(Siddique and Qureshi, 2020).** This medium was used for the cultivation of actinobacterial isolates and the synthesis of secondary metabolites

| Components | (g/L) |
| --- | --- |
| Soluble corn starch | 50.0 |
| KCl | 0.1 |
| NaCl | 0.5 |
| Yeast extract | 2.0 |
| MgSO_4_.7H_2_O | 0.1 |
| CaCO_3_ | 0.8 |
| α-amylase | 0.1 |
| pH | 7.2 ± 0.2 |
| Shake flask experiments were carried out in 250 ml Erlenmeyer containing 50 ml of production medium, inoculated with 5 ml (10% v/v) of seed medium separately and incubated in a 150-rpm rotary shaker at 28°C for 10 days as described earlier by **Siddique *et al.* (2013).** | |

**Table S3** The Taguchi design’s experimental matrix for the selection of the significant variables for abamectin synthesis and mycelium production by the *S. avermitilis*

| **Run no.** | **Variables** | | | | | | |
| --- | --- | --- | --- | --- | --- | --- | --- |
|  | **A** | **B** | **C** | **D** | **E** | **E** | **G** |
| **1** | +1 | -1 | +1 | -1 | +1 | -1 | +1 |
| **2** | -1 | +1 | +1 | -1 | -1 | +1 | +1 |
| **3** | +1 | +1 | -1 | +1 | -1 | -1 | +1 |
| **4** | +1 | +1 | -1 | -1 | +1 | +1 | -1 |
| **5** | -1 | -1 | -1 | -1 | -1 | -1 | -1 |
| **6** | -1 | -1 | -1 | +1 | +1 | +1 | +1 |
| **7** | +1 | -1 | +1 | +1 | -1 | +1 | -1 |
| **8** | -1 | +1 | +1 | +1 | +1 | -1 | -1 |

**Table S4** The experimental matrix of Box-Behnken's design for the maximization of abamectin synthesis and mycelium production in the *S. avermitilis*

| **Run no.** | **Variables** | | |
| --- | --- | --- | --- |
|  | **A** | **B** | **C** |
| 1 | +1 | 0 | -1 |
| 2 | 0 | 0 | 0 |
| 3 | 0 | 0 | 0 |
| 4 | -1 | 0 | -1 |
| 5 | 0 | -1 | +1 |
| 6 | -1 | 0 | +1 |
| 7 | +1 | 0 | +1 |
| 8 | 0 | +1 | +1 |
| 9 | +1 | +1 | 0 |
| 10 | 0 | 0 | 0 |
| 11 | 0 | +1 | -1 |
| 12 | 0 | 0 | 0 |
| 13 | 0 | 0 | 0 |
| 14 | -1 | -1 | 0 |
| 15 | 0 | -1 | -1 |
| 16 | -1 | +1 | 0 |
| 17 | +1 | -1 | 0 |

**Table S5** Abamectin B1b and B1a quantification using HPLC analysis

| **Samples** | **Peak** | **Ret. Time** | **Area** | **Concentration** | **Unit** | **Name** |
| --- | --- | --- | --- | --- | --- | --- |
| Standard abamectin | 1 | 1.421 | 250.13 | 0.016 | µg/5µl | Abamectin B1b |
|  | 2 | 2.173 | 1009 | 0.064 | µg/5µl | Abamectin B1a |
|  | Total |  | 1259.13 |  |  |  |
| *S. avermitilis* (cell extract) growth on starch nitrate medium after fifteen days of incubation | 1 | 1.421 | 1333.38 | 0.084 | µg/5µl | Abamectin B1b |
|  | 2 | 2.173 | 7150 | 5.391 | µg/5µl | Abamectin B1a |
|  | Total |  | 8483.38 |  |  |  |
| *S. avermitilis* (cell extract) growth on Production medium after twelve days of incubation | 1 | 1.421 | 6988.16 | 4.439 | µg/5µl | Abamectin B1b |
|  | 2 | 2.173 | 18462 | 11.731 | µg/5µl | Abamectin B1a |
|  | Total |  | 25450.16 |  |  |  |
| *S. avermitilis* (cell extract) growth on maximization medium after ten days of incubation | 1 | 1.421 | 19723.23 | 12.511 | µg/5µl | Abamectin B1b |
|  | 2 | 2.173 | 45600 | 28.994 | µg/5µl | Abamectin B1a |
|  | Total |  | 65325.23 |  |  |  |
