# Supplementary material for: Optimization of abamectin production by Streptomyces avermitilis and its antagonistic activity against Meloidogyne incognita
Source: BMC Biotechnol. 2026 Mar 11;26:41. doi: 10.1186/s12896-026-01112-6 (PMC13064350; doi:10.1186/s12896-026-01112-6)
Supplement: Supplementary file 2 — Supplementary Material 2 [file 12896_2026_1112_MOESM2_ESM.docx]

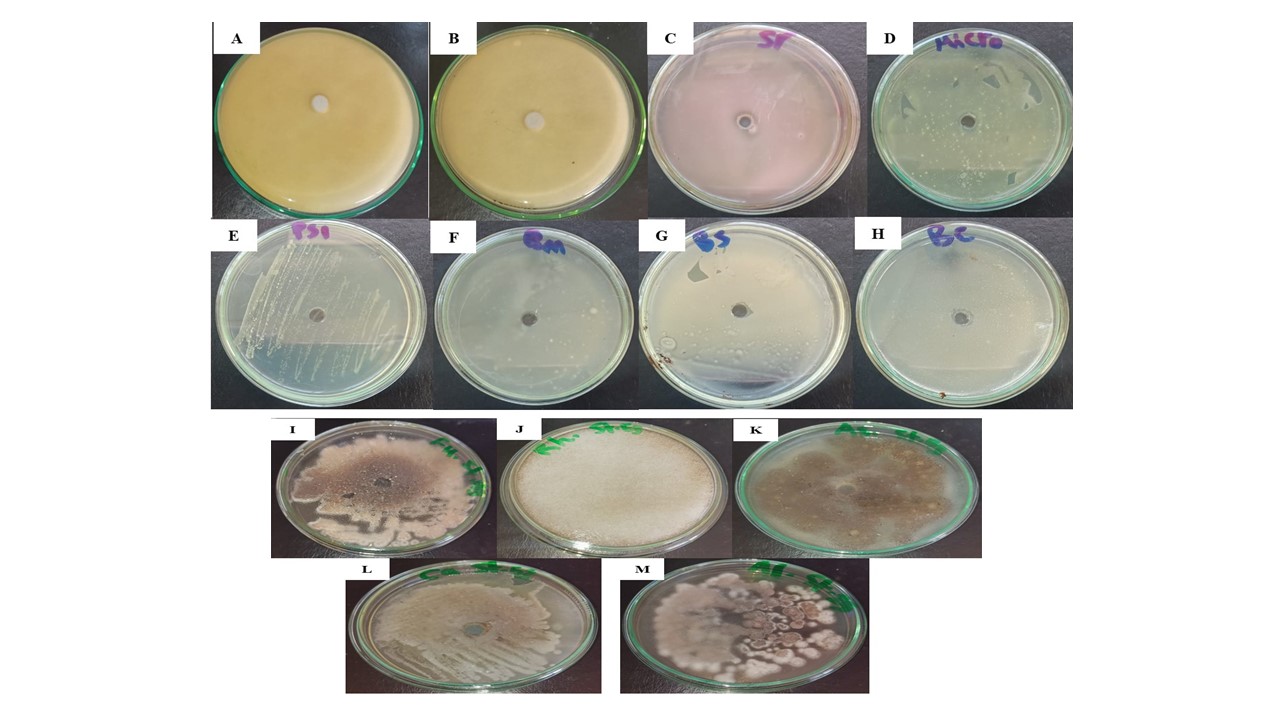


**Fig.** **S1** Antimicrobial activities of *S. avermitilis* against bacterial species using the well-diffusion method on Muller-Hinton agar medium: **(A)** *Erwinia carotovora* **(B)** *Xanthomonas campestris* **(C)** *Serratia marcescens* **(D)** *Micrococcus luteus* **(E)** *Pseudomonas fluorescens* **(F)** *Bacillus megaterium* **(G)** *Bacillus subtilis* **(H)** *Bacillus circulans* and fungal species: **(I)** *Fusarium oxysporum* **(J)** *Rhizopus nigricans* **(K)** *Aspergillus niger* **(L)** *Candida albicans* and **(M)** *Alternaria solani*


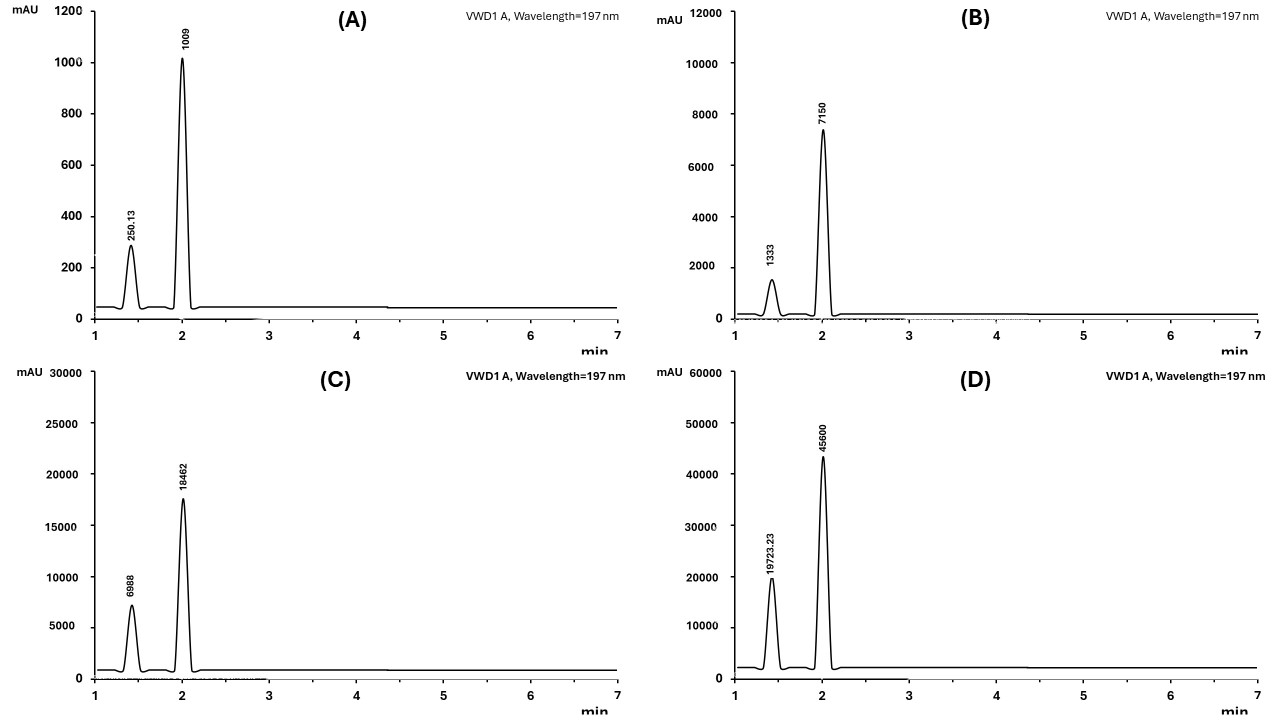


**Fig. S2** **(A)** Standards of abamectin B1b and B1a by HPLC analysis, **(B)** Quantification of abamectin B1b and B1a produced by *S. avermitilis* (cell extract) growth on starch nitrate medium after fifteen days of incubation using HPLC analysis, **(C)** Quantification of abamectin B1b and B1a produced by *S. avermitilis* (cell extract) growth on Production medium after twelve days of incubation using HPLC analysis, **(D)** Quantification of abamectin B1b and B1a produced by *S. avermitilis* (cell extract) growth on maximization medium after ten days of incubation using HPLC analysis.
